# *Chamaecyparis obtusa* Suppresses Virulence Genes in* Streptococcus mutans*

**DOI:** 10.1155/2016/2396404

**Published:** 2016-05-11

**Authors:** Eun-Hee Kim, Sun-Young Kang, Bog-Im Park, Young-Hoi Kim, Young-Rae Lee, Jin-Hee Hoe, Na-Young Choi, Ji-Young Ra, So-Youn An, Yong-Ouk You

**Affiliations:** ^1^Department of Pediatric Dentistry, School of Dentistry, Wonkwang University, Iksan 54538, Republic of Korea; ^2^Department of Oral Biochemistry, School of Dentistry, Wonkwang University, Iksan 54538, Republic of Korea; ^3^Department of Food Science and Technology, College of Agriculture and Life Science, Chonbuk National University, Jeonju 54896, Republic of Korea; ^4^APLUS AROMA, Iksan 54627, Republic of Korea; ^5^College of Education, Wonkwang University, Iksan 54538, Republic of Korea; ^6^Wonkwang Research Institute for Food Industry, Iksan 54538, Republic of Korea

## Abstract

*Chamaecyparis obtusa (C. obtusa)* is known to have antimicrobial effects and has been used as a medicinal plant and in forest bathing. This study aimed to evaluate the anticariogenic activity of essential oil of* C. obtusa* on* Streptococcus mutans*, which is one of the most important bacterial causes of dental caries and dental biofilm formation. Essential oil from* C. obtusa* was extracted, and its effect on bacterial growth, acid production, and biofilm formation was evaluated.* C. obtusa* essential oil exhibited concentration-dependent inhibition of bacterial growth over 0.025 mg/mL, with 99% inhibition at a concentration of 0.2 mg/mL. The bacterial biofilm formation and acid production were also significantly inhibited at the concentration greater than 0.025 mg/mL. The result of LIVE/DEAD® BacLight*™* Bacterial Viability Kit showed a concentration-dependent bactericidal effect on* S. mutans* and almost all bacteria were dead over 0.8 mg/mL. Real-time PCR analysis showed that gene expression of some virulence factors such as* brpA, gbpB, gtfC,* and* gtfD* was also inhibited. In GC and GC-MS analysis, the major components were found to be *α*-terpinene (40.60%), bornyl acetate (12.45%), *α*-pinene (11.38%), *β*-pinene (7.22%), *β*-phellandrene (3.45%), and *α*-terpinolene (3.40%). These results show that* C. obtusa* essential oil has anticariogenic effect on* S. mutans*.

## 1. Introduction

Dental caries is the most common infectious oral disease that has afflicted humans including children and adolescents [1]. It is a multifactorial disease, which is caused by detrimental changes in bacterial ecology due to formation of a biofilm that adheres to the tooth surface [2]. During the past few decades, many reports worldwide showed an overall decreasing trend of dental caries. However, recent studies have reported an alarming increase in caries prevalence, especially among the underprivileged groups [3].

*S. mutans* can colonize the oral cavity and form bacterial biofilm. It has the ability to survive in an acidic environment and interact with other microorganisms colonizing this ecosystem [2].

Caries results from an imbalance between demineralization and remineralization of tooth structure. Acidogenic bacteria ferment dietary carbohydrates, thereby producing organic acids, which initiate dissolution of tooth enamel and breakdown of dental tissue [4]. The extent of the pH fall is influenced by numerous factors, including the composition of the microflora, as well as the type and frequency of sugar intake [5].

*S. mutans* produce glucosyltransferase (GTF) enzyme which is recognized as virulence factors in the etiology of dental caries. GTF enzymes synthesize extracellular glucans and contribute significantly to the dental plaque matrix's polysaccharide formation [6]. The sucrose-dependent mechanism of plaque formation is based on GTF produced by* S. mutans* in combination with glucan-binding proteins (GBPs). The synthesized glucans provide the possibility of both bacterial adhesion to the tooth enamel and adhesion of the microorganisms to each other [2].

Demineralization can be reversed by calcium and phosphate, together with fluoride, diffusing into the tooth and depositing a new veneer on the crystal remnants in the noncavitated lesion, and is known as remineralization [4]. Fluoride has been used as the “first choice” for the prevention of dental caries [7], and other anticariogenic natural products or compounds like xylitol have also been introduced [8].

*C. obtusa* is a tropical tree species found in Japan and the southern region of South Korea, and essential oil is extracted from leaves and twigs of the* C. obtusa* tree. The essential oil has several types of terpenes and has been commercially used in soaps, toothpaste, and cosmetics as a functional additive [9]. The essential oil of* C. obtusa* is a concentrated hydrophobic liquid containing volatile compound with natural antibiotic properties that protect against harmful insects, animals, and microorganisms. Inhalation of this essential oil is known as* C. obtusa* aromatherapy or* C. obtusa* forest bathing [10] and has been shown to exert antibacterial and antifungal effects [11].

This study was performed to analyze anticariogenic effect of* C. obtusa* on* S. mutans* and to determine its chemical composition using a gas chromatography (GC)/gas chromatography-mass spectrometry (GC-MS) analysis.

## 2. Materials and Methods

### 2.1. Plant Material and Essential Oil

*C. obtusa* was collected in October 2013 from the Jeollanam-do province, South Korea. Fresh leaves and twigs of* C. obtusa* (1 kg) were ground mechanically and hydrodistilled for 3 hours using a Clevenger-type apparatus. The yield of the* C. obtusa* essential oil was 1.08% of yellow pale oil, based on the fresh weight of the plant. The* C. obtusa* essential oil was stored in a deep freezer (−70°C) to minimize the loss of volatile compounds.

### 2.2. Inhibition of Bacterial Growth

*S. mutans* (ATCC 25175) was purchased from the American Type Culture Collection (ATCC; Rockville, MD, USA) and cultured in brain heart infusion (BHI; Difco, Detroit, MI, USA) broth under aerobic condition at 37°C. The growth of* S. mutans* was examined at 37°C in 0.95 mL of brain heart infusion broth containing various concentrations of the* C. obtusa*. These tubes were inoculated with 0.05 mL of an overnight culture grown in the BHI broth (final: 5 × 10^5^ colony-forming units [CFU]/mL) and incubated at 37°C for 24 h. The optical density (OD) of cells was measured at 550 nm using a spectrophotometer. Each concentration of the extract was tested in triplicate.

### 2.3. Acid Production

Acid production by* S. mutans* was examined to evaluate the effect of* C. obtusa* essential oil using a modification of previously described method [12]. The* C. obtusa* essential oil was filter-sterilized using membrane filter with 0.2 *μ*m pore size and added to 0.95 mL of the phenol red broth containing 1% glucose, which was then inoculated with 0.05 mL of the seed culture of* S. mutans*. After 24 h of cultivation, the pH of the cultures was determined using a pH meter (Corning, Inc., Corning, NY, USA). Three replicates were measured for each concentration of the test extract.

### 2.4. Biofilm Formation Assay

The biofilm assay was based on a previously described method [13, 14]. Biofilm formation was measured by staining with safranin and observed by scanning electron microscopy (SEM).* C. obtusa* essential oil was added to BHI broth containing 0.1% sucrose in 35 mm polystyrene dishes. The cultures were then inoculated with a seed culture of* S. mutans* (final: 5 × 10^5^ CFU/mL) and incubated for 24 h at 37°C. After incubation, the supernatants were removed, and the dishes were rinsed with distilled water. Biofilm formation were stained with 0.1% safranin and photographed. The bound safranin was released from the stained cells with 30% acetic acid and the absorbance of the solution was measured at 530 nm. In addition, biofilm on 35 mm polystyrene dishes was observed by SEM [15]. The biofilm formed on the dishes was rinsed with distilled water, fixed with 2.5% glutaraldehyde in 0.1 M sodium cacodylate buffer (pH 7.2) at 4°C for 24 h, and dehydrated with ethanol gradient series (60%, 70%, 80%, 90%, 95%, and 100%). Then, the samples were freeze-dried, sputter-coated with gold (108A sputter coater; Cressington Scientific Instruments, Inc., Watford, England, United Kingdom), and observed by SEM (JSM-6360, JEOL, Tokyo, Japan).

### 2.5. Confocal Laser Scanning Microscopy

To determine the bactericidal effect of* C. obtusa* essential oil on* S. mutans*, staining with LIVE/DEAD®* Bac*Light*™* Bacterial Viability Kit was performed and examined by confocal laser scanning microscopy. The cultured* S. mutans* in BHI was diluted using BHI media to 1 × 10^7^ CFU/mL and treated with the essential oil at 37°C under aerobic conditions. After 30 min of incubation, the bacteria were washed with PBS and stained with the LIVE/DEAD®* Bac*Light*™* Bacterial Viability Kit (Molecular Probes, Eugene, OR, USA), prepared according to the manufacturer's protocol. After 15 min of staining, bacteria were observed using confocal laser scanning microscopy (LSM 510, Zeiss, Oberkochen, Germany).

### 2.6. Real-Time Polymerase Chain Reaction Analysis

A real-time PCR was performed to examine the effect of* C. obtusa* essential oil on virulence factor gene expression of* S. mutans.* The subminimal inhibitory concentration (0.025–0.1 mg/mL) of the essential oil was used. After 24 h of culture, total RNA was isolated from* S. mutans* using a TRIzol® Reagent (Life Technologies, Carlsbad, CA, USA) and cDNA was synthesized. The amplification was performed using a StepOnePlus Real-Time PCR system with SYBR® Green Master Mix (Applied Biosystems, Carlsbad, CA, USA). 16S rRNA was used as an internal control.

### 2.7. GC and GC-MS Analysis

GC analysis was performed on a Hewlett-Packard model 6890 series gas chromatograph with a flame ionization detector (FID) and a split ratio of 30 : 1 using DB-5HT fused silica capillary columns (30 m × 0.32 mm, i.d., 0.10 *μ*m film thickness). The temperature of column was programmed from 40°C to 230°C, at 2°C/min and then kept constant at 230°C for 20 min. The injector and detector temperatures were 230°C and 250°C, respectively. The gas carrier used was nitrogen, at a flow rate of 0.80 mL/min. Peak areas were measured by electronic integration and the relative amounts of the individual components were determined based on the peak areas. The GC-MS analysis was carried out on an Agilent Technologies 7890A GC and 5975C mass selective detector (MSD) operating in EI mode at 70 eV, fitted with a DB-5MS fused silica capillary column (30 m × 0.25 mm, i.d., 0.25 *μ*m film thickness). The column temperature was programmed from 40°C to 230°C at 2°C/min and then kept constant at 230°C for 20 min. The injector and ion source temperatures were 250°C, respectively. The gas carrier was helium at a flow rate of 1.0 mL/min. The identification of individual components was based on comparisons with Wiley 7n/NIST 05 mass spectra libraries and retention indices with reference to literature data. Linear retention indices were calculated against those of n-paraffin (C6–C26) series [16].

### 2.8. Statistical Analysis

All experiments were performed in triplicate. Data were analyzed using SPSS software 12.0 (Chicago, IL, USA). The data are expressed as the mean ± standard deviation values. The statistical analysis was done using Student's *t*-test. Values of *p* < 0.01 were considered statistically significant.

## 3. Results

### 3.1. Inhibition of Bacterial Growth

**Figure 1:**
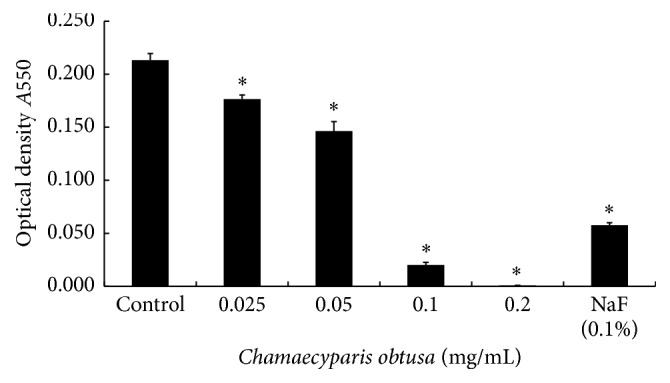
Bacterial growth inhibition of* Streptococcus mutans* (*S. mutans*) by* Chamaecyparis obtusa* (*C. obtusa*) essential oil. The optical density (*A*550) was read using a spectrophotometer. ^*∗*^ *p* < 0.01 compared to the control group.

After extraction of* C. obtusa* essential oil by hydrodistillation, the antibacterial activity of the oil was tested against* S. mutans*.* C. obtusa* essential oil significantly inhibited the growth of* S. mutans* in a concentration-dependent manner. The bacteria were treated with 0.025, 0.05, 0.1, and 0.2 mg/mL of* C. obtusa* essential oil. When treated with 0.025 mg/mL of the essential oil, the bacterial growth was significantly inhibited in comparison to the control group (*p* < 0.05). The positive control (0.1% NaF) also showed antibacterial activity (Figure 1).

### 3.2. Inhibition of Acid Production

To investigate whether* C. obtusa* essential oil can inhibit* S. mutans* organic acid production, the bacteria were cultured in the presence of various concentrations (0.025–0.2 mg/mL) of the essential oil and the change in pH was measured. The pH of the control declined to 5.30 after bacterial culture, while the initial pH of the media before bacterial culture was 7.40. However, the addition of 0.025, 0.05, 0.1, and 0.2 mg/mL of* C. obtusa* essential oil resulted in pH levels of 5.38, 5.73, 7.12, and 7.40, respectively. These results indicate that* C. obtusa* essential oil may inhibit the organic acid production by* S. mutans.* NaF (0.1%) used for the positive control also inhibited acid production, resulting in a pH of 7.10 (Table 1).

### 3.3. Inhibition of Biofilm Formation

**Figure 2:**
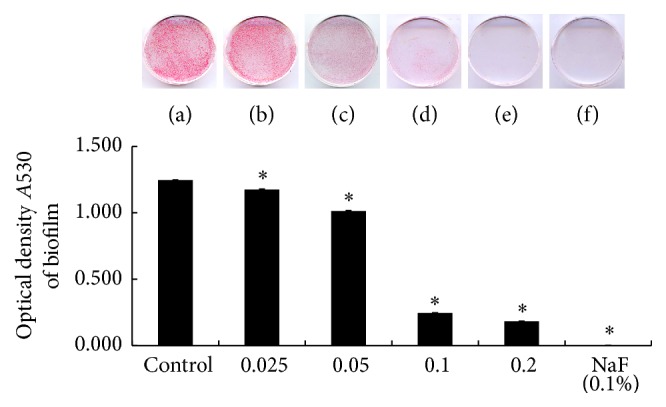
Inhibitory effect of* C. obtusa* essential oil on biofilm formation by* S. mutans*, as observed by safranin staining. (a) Control; (b) 0.025 mg/mL; (c) 0.05 mg/mL; (d) 0.1 mg/mL; (e) 0.2 mg/mL essential oil of* C. obtusa*; and (f) positive control (0.1% NaF). ^*∗*^Significance was determined at ^*∗*^ *p* < 0.01 compared to the control group.

**Figure 3:**
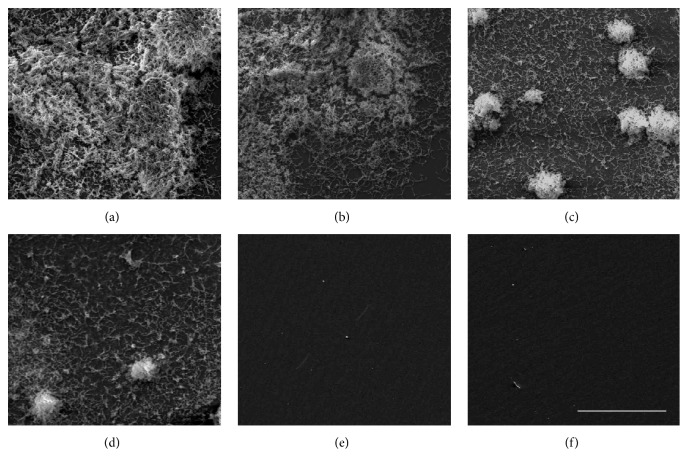
Inhibitory effect of* C. obtusa* essential oil on biofilm formation by* S. mutans*, as observed by scanning electron microscopy. (a) Control; (b) 0.025 mg/mL; (c) 0.05 mg/L; (D) 0.1 mg/mL; (e) 0.2 mg/mL; and (f) positive control (0.1% NaF). Scale bar = 10 *μ*m.

To determine whether* C. obtusa* essential oil inhibits biofilm formation by* S. mutans*, the bacteria were cultured in the presence of various concentration of* C. obtusa* essential oil in polystyrene dishes. Biofilm formation was studied using safranin staining, and absorbance was measured at 530 nm. The biofilm formation by* S. mutans* was significantly inhibited by treatment with* C. obtusa* essential oil in a dose-dependent manner over 0.025 mg/mL of* C. obtusa* essential oil. When treated with 0.1% NaF (positive control), complete inhibition was shown (Figure 2). SEM results were consistent with those of safranin staining (Figure 3).

### 3.4. Bactericidal Effect

**Figure 4:**
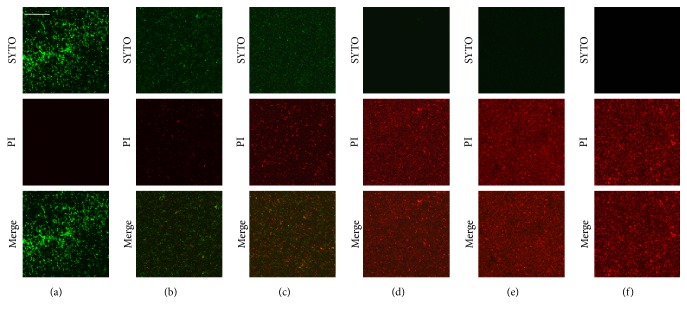
Bactericidal effect of* C. obtusa* essential oil. Cultured* S. mutans* was treated with* C. obtusa* extract (0.2–1.6 mg/mL) and stained with LIVE/DEAD®* Bac*Light*™* Bacterial Viability Kit. The stained bacteria were observed by confocal laser scanning microscopy. Treatment with* C. obtusa* decreased green-labeled living bacteria (SYTO® 9 stain) and increased red-labeled dead bacteria (PI stain) in a dose-dependent manner. (a) Control; (b) 0.2 mg/mL; (c) 0.4 mg/mL; (d) 0.8 mg/mL; (e) 1.6 mg/mL; and (f) positive control (0.1% NaF). Bar = 100 *μ*m.

To evaluate bactericidal effect of* C. obtusa* essential oil,* S. mutans* were cultured in presence of high concentrations (0.2–1.6 mg/mL) of the essential oil, stained with LIVE/DEAD®* Bac*Light*™* Bacterial Viability Kit and observed using confocal laser scanning microscopy. Treatment with* C. obtusa* essential oil decreases living bacteria (green fluorescence labeled cell stained by SYTO® 9) and increases dead bacteria (red fluorescence labeled cell stained by PI). The bactericidal effect of* C. obtusa* essential oil was also observed in a dose-dependent manner (Figure 4). However, low concentration (lesser than 0.2 mg/mL) of the essential oil did not show bactericidal effect (data not shown).

### 3.5. Inhibition of Virulence Factor Gene Expression

**Figure 5:**
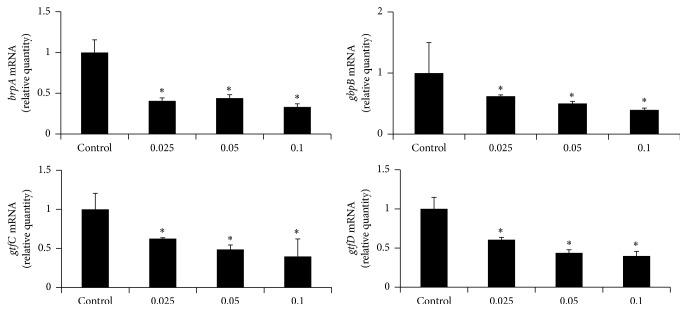
Inhibitory effect of* C. obtusa* essential oil on virulence factor gene expression.* S. mutans* was cultured and treated with subminimal inhibitory concentration (0.025–0.1 mg/mL) of* C. obtusa* extract, and real-time polymerase chain reaction (PCR) analysis was performed. The* brpA, gbpB, gtfC,* and* gtfD* expressions were significantly inhibited at 0.025 mg/mL of* C. obtusa*. Each value is expressed as mean ± standard deviation. ^*∗*^Significance was determined at ^*∗*^ *p* < 0.01 when compared with the control group.

To evaluate the influence of* C. obtusa* on the gene expression of virulence factors in* S. mutans*, the bacteria were treated with subminimal inhibitory concentration (0.025–0.1 mg/mL) of* C. obtusa*, and the gene expressions of various virulence factors were assessed by real-time PCR. We evaluated the genetic expressions of* brpA*,* gbpB*,* gtfC*, and* gtfD*. The expressions of* brpA*, which contribute to the control of the phosphotransferase system (PTS) and are related with acid tolerance, and* gbpB* which contribute to bacterial adherence, were significantly decreased at 0.025 mg/mL of* C. obtusa*. The expression of* gtfC* and* gtfD* which encode GTFase C and D proteins was significantly decreased when* S. mutans* was treated with 0.025 mg/mL of* C. obtusa* (Figure 5).

### 3.6. The Composition of* C. obtusa* Essential Oil by GC and GC-MS Analysis

Fifty-nine constituents were identified in the* C. obtusa* essential oil, accounting for up to 97.38% of the total material. Thus, all unidentified compounds were only minor components. The major components included *α*-terpinene (40.60%), bornyl acetate (12.45%), *α*-pinene (11.38%), *β*-pinene (7.22%), *β*-phellandrene (3.45%), and *α*-terpinolene (3.40%) (Table 2).

## 4. Discussion

Fluoride plays an important role in the prevention and control of dental caries. However, an unfortunate side effect of fluoride is fluorosis. Ingestion of fluoride before 2 to 3 years of age is considered critical for possible fluorosis in the permanent dentition [17]. Therefore, natural products are currently receiving special attention as a good alternative to synthetic chemical substances for the prevention of dental caries [18].

Essential oils are volatile components mainly obtained by distillation of plant and consist of a mixture of various terpenoids. Terpenes are the therapeutic chemical substances present in medicinal plants [19]. Essential oils extracted from various plants are known to have antimicrobial activity [20]. Some natural derivatives like extracts from* Myristica fragrans*,* Lippia sidoides*,* Hyptis pectinata*,* Curcuma longa*, and* Baccharis dracunculifolia* have been proved to be effective against* S. mutans* [21–25]. This study was performed to evaluate anticariogenic activity of* C. obtusa* essential oil on* S. mutans*.

To evaluate anticariogenic properties of* C. obtusa* essential oil,* S. mutans* was used because these bacteria is considered as a major cause for the formation of dental caries [2]. Our results showed that growth of* S. mutans* was suppressed by treatment with* C. obtusa* essential oil. Furthermore, the results of LIVE/DEAD®* Bac*Light*™* Bacterial Viability Kit also showed that* C. obtusa* essential oil has a bactericidal effect against* S. mutans*. These results suggested that* C. obtusa* essential oil has a potential for anticariogenic effect, which is interesting since the inhibition of the growth of* S. mutans* is one of the strategies for prevention of dental caries.

Growth of* S. mutans* was suppressed by treatment with* C. obtusa* essential oil in a concentration-dependent manner above the concentration of 0.025 mg/mL. In dental plaque formation, pH is one of the major factors, since low pH leads to demineralized hydroxyapatite and favors the cariogenicity [26].* S. mutans* can metabolize dietary sugars and produce organic acid. Low-pH environment in the biofilm matrix results in dissolution of enamel, thus initiating the pathogenesis of dental caries. Therefore, the alteration of pH is used as an indicator to determine the effect of anticariogenic agents [27]. In this study,* C. obtusa* essential oil inhibited the decrease of pH induced by* S. mutans* and the result suggests that* C. obtusa* essential oil may inhibit dental caries through inhibition of acid production by* S. mutans*.

Biofilms are communities of microorganisms that adhere to biological or abiotic substrata and produce an extracellular matrix typically comprising of polysaccharides and proteins. Dental plaque is a kind of biofilm found on a tooth surface, embedded in a matrix of host and bacterial polymers [28]. In biofilm, known as plaque in the oral cavity, the interaction of specific bacteria with constituents of the diet results in caries [27]. Biofilm formation by* S. mutans* was also inhibited by treatment with* C. obtusa* essential oil cultured on polystyrene dishes. These results suggested that* C. obtusa* essential oil directly inhibits the biofilm formation by* S. mutans*.

Furthermore, several virulence gene factors of* S. mutans* are associated with various aspects of cariogenicity such as acid tolerance, bacterial adhesion, and biofilm formation [2]. The* brpA* has been shown to contribute to biofilm formation and plays a major role in cell envelope biogenesis/homeostasis and regulation of stress response as well as in acid tolerance [29, 30]. The g*bpB*, which encodes surface-associated glucan-binding protein (GBP), mediates binding of bacteria to glucans and enables development of biofilm. The g*tfC* and g*tfD* encode glucosyltransferases (GTFase) which are essential virulence factor in plaque development and are responsible for glucans formation from sucrose [2]. In this study, to evaluate correlation between inhibitory effect by* C. obtusa* essential oil and virulence factors expression, we determined the mRNA expression level of several virulence factors using a real-time PCR analysis. We evaluated the gene expression level of* brpA*,* gbpB*,* gtfC*, and* gtfD. C. obtusa* essential oil significantly inhibited the transcription level of* brpA*,* gbpB*,* gtfC*, and* gtfD*.

Based on our results of GC/GC-MS analysis, the major components included *α*-terpinene (40.60%), bornyl acetate (12.45%), *α*-pinene (11.38%), *β*-pinene (7.22%), *β*-phellandrene (3.45%), and *α*-terpinolene (3.40%) (Table 2). Although the biological activities of* C. obtusa* essential oil are not yet fully understood, some previous study reported that several types of terpenes of* C. obtusa* have been shown to exert antibacterial and antifungal effect [11]. Recent studies also reported other beneficial properties of* C. obtusa*, such as pharmacological activities for the treatment of atopic dermatitis [31], improvement on cognitive function of the central nervous system on rat experimental model [10], and promotion of hair growth [32]. In addition,* C. obtusa* has antinociceptive and anti-inflammatory properties, which increases its applicability in oral care [9, 33].

Based on our results, we conclude that* C. obtusa* may have a possible practical use against the cariogenic bacteria within the mouth and recommend further investigation of* C. obtusa* on periodontopathic and cariogenic bacteria.

## 5. Conclusions

This study has proved that* C. obtusa* essential oil exhibited significant inhibition of bacterial growth, acid production, and biofilm formation by* S. mutans*. Also* C. obtusa* essential oil showed bactericidal effect. Furthermore,* C. obtusa* essential oil also inhibited the transcription level of several virulence factors such as* brpA*,* gbpB*,* gtfC*, and* gtfD* of* S. mutans.* In GC and GC-MS analysis, the major components were *α*-terpinene (40.60%), bornyl acetate (12.45%), *α*-pinene (11.38%), *β*-pinene (7.22%), *β*-phellandrene (3.45%), and *α*-terpinolene (3.40%). Therefore, the results of this study indicate that* C. obtusa* essential oil showed good anticariogenic effect on* S. mutans* and appear to be a promising new agent that may prevent dental caries. Further studies are needed to develop the new medicine for clinical use.

## Figures and Tables

**Table 1:** Inhibitory effect of *C. obtusa* essential oil on acid production by *S. mutans*.

Con. (mg/mL)	pH (before incubation)	pH (after incubation)
Control	7.40 ± 0.00	5.30 ± 0.00
0.025	7.40 ± 0.00	5.38 ± 0.01^*∗*^
0.05	7.39 ± 0.00	5.73 ± 0.00^*∗*^
0.1	7.40 ± 0.00	7.12 ± 0.00^*∗*^
0.2	7.40 ± 0.00	7.40 ± 0.00^*∗*^
0.1% NaF	7.40 ± 0.00	7.10 ± 0.00^*∗*^

Data (pH) are represented as mean ± standard deviation.

^*∗*^ *p* < 0.01 when compared with the control group after incubation.

**Table 2:** Gas chromatography and gas chromatography-mass spectrometry (GC/GC-MS) analysis of the essential oil isolated from* C. obtusa*.

Retention time (min)	Retention index^a^	Compound	Peak area (%)
8.849	801	n-Hexanal	0.18
11.982	852	*trans*-2-Hexenal	0.28
13.110	870	n-Hexanol	0.06
15.125	902	Bornylene	0.12
16.419	919	Tricyclene	0.77
16.763	925	*α*-Thujene	0.12
17.742	936	*α*-Pinene	11.38
18.650	952	Camphene	2.87
20.228	975	Sabinene	0.46
21.345	980	*δ*-3-Carene	0.37
22.047	983	*β*-Pinene	7.22
24.483	1023	*α*-Terpinene	40.60
24.927	1029	Limonene	0.57
25.474	1036	*β*-Phellandrene	3.45
27.317	1061	*γ*-Terpinene	0.21
28.393	1072	*cis*-Sabinene hydrate	0.35
29.742	1089	*α*-Terpinolene	3.40
29.983	1092	Dehydro-*p*-cymene	0.53
30.874	1102	*trans*-Sabinene hydrate	0.09
31.769	1115	1-Octen-3-yl acetate	0.06
33.199	1133	*trans*-*p*-2-Menthen-1-ol	0.05
36.312	1173	Cryptone	0.11
36.461	1175	p-Cymen-8-ol	0.10
36.965	1181	Terpinen-4-ol	0.23
37.889	1193	*α*-Terpineol	0.11
38.960	1207	Verbenone	0.09
39.699	1217	Fenchyl acetate	0.07
42.535	1256	Linalyl acetate	0.08
45.552	1296	Bornyl acetate	12.45
49.502	1352	*α*-Terpinyl acetate	0.62
51.422	1380	*α*-Copaene	0.28
53.780	1414	*α*-Cedrene	0.52
54.015	1417	*β*-Caryophyllene	0.16
54.331	1422	*β*-Cedrene	0.25
55.009	1431	*cis*-Thujopsene	0.07
55.419	1434	*β*-Gurjunene	0.06
56.378	1453	*α*-Humulene	0.37
56.976	1462	*cis*-Muurola-4(14),5-diene	0.24
57.656	1472	*β*-Cadinene	0.06
57.927	1476	Germacrene D	0.13
58.150	1490	*β*-Himachalene	0.10
59.003	1492	*α*-Muurolene	0.16
59.519	1497	*α*-Chamigrene	0.05
60.024	1508	*γ*-Cadinene	0.38
61.079	1525	*δ*-Cadinene	0.15
61.522	1530	Cadina-1,4-diene	0.09
61.937	1539	Selina-3,7(11)-diene	0.07
63.011	1551	Elemol	0.07
63.406	1562	*trans*-Nerolidol	0.25
64.651	1578	Spathulenol	0.05
64.916	1586	Caryophyllene oxide	0.10
66.435	1603	Cedrol	0.97
67.079	1622	1-*epi*-Cubenol	0.08
67.835	1635	*γ*-Eudesmol	0.58
68.123	1239	*τ*-Cadinol	0.57
68.964	1648	*α*-Cadinol	0.09
69.973	1670	Bulnesol	0.07
70.829	1685	*α*-Bisabolol	0.13
82.676	1898	Rimuene	0.27
84.836	1926	Hibaene	2.33
85.085	1945	Pimaradiene	0.10
86.747	1977	13-Isopimaradiene	1.42
88.068	1998	Dolabradiene	0.09
90.446	2051	Dehydroabietane	0.07

Total			97.38

^a^Retention index on DB-5HT column.
